# Immune Vulnerability of Infants to Tuberculosis

**DOI:** 10.1155/2013/781320

**Published:** 2013-05-13

**Authors:** Koen Vanden Driessche, Alexander Persson, Ben J. Marais, Pamela J. Fink, Kevin B. Urdahl

**Affiliations:** ^1^Centre for Understanding and Preventing Infections in Children, Child & Family Research Institute, University of British Columbia, Vancouver, BC, Canada V5Z 4H4; ^2^Department of Pediatrics, Laboratory of Pediatric Infectious Diseases, Radboud University Medical Centre, 6500 HB Nijmegen, The Netherlands; ^3^Sydney Institute for Emerging Infectious Diseases and Biosecurity and The Children's Hospital at Westmead, University of Sydney, Locked Bag 4100, Sydney, NSW 2145, Australia; ^4^Department of Immunology, University of Washington, Seattle, WA 98195, USA; ^5^Seattle Biomedical Research Institute, Seattle, WA 98109, USA; ^6^Department of Pediatrics, University of Washington, Seattle, WA 98195, USA

## Abstract

One of the challenges faced by the infant immune system is learning to distinguish the myriad of foreign but nonthreatening antigens encountered from those expressed by true pathogens. This balance is reflected in the diminished production of proinflammatory cytokines by both innate and adaptive immune cells in the infant. A downside of this bias is that several factors critical for controlling *Mycobacterium tuberculosis* infection are significantly restricted in infants, including TNF, IL-1, and IL-12. Furthermore, infant T cells are inherently less capable of differentiating into IFN-**γ**-producing T cells. As a result, infected infants are 5–10 times more likely than adults to develop active tuberculosis (TB) and have higher rates of severe disseminated disease, including miliary TB and meningitis. Infant TB is a fundamentally different disease than TB in immune competent adults. Immunotherapeutics, therefore, should be specifically evaluated in infants before they are routinely employed to treat TB in this age group. Modalities aimed at reducing inflammation, which may be beneficial for adjunctive therapy of some forms of TB in older children and adults, may be of no benefit or even harmful in infants who manifest much less inflammatory disease.

## 1. Introduction

It is believed that one third of the world population is infected with *Mycobacterium tuberculosis *(*M.tb*) [1]. However, the majority of adults have immune systems capable of containing *M.tb* without developing active disease even if they are unable to completely eradicate the organism from their bodies. These individuals are said to be latently infected although “latent infection” is now appreciated to be a highly dynamic state. Latently infected individuals are asymptomatic, but harbor a 5–10% lifetime risk of developing active disease [2]. Of those individuals who eventually progress to active tuberculosis (TB), approximately half will do so within 2 years of acquiring the infection [3] although reactivation can occur even 30 or more years after primary infection [4]. In endemic countries, TB acquired later in life is more often due to reinfection with another *M.tb* strain than to reactivation of latent infection [5, 6]. Overall, no bacterial organism in the world claims more casualties than *M.tb*, with an estimated 8.7 million new cases and 1.4 million deaths in 2011 [7].

The number of pediatric deaths attributable to TB is harder to estimate because TB in children is difficult to diagnose, especially in resource-limited settings carrying the greatest burden of disease [8]. This is likely the explanation why UNICEF does not include TB in its under-five mortality reports [9]. It is apparent from natural history of disease studies conducted in the prechemotherapy era that *M.tb*-infected infants (children less than one year of age) are at much greater risk for progression to active TB than are immunocompetent adults. A recent nosocomial TB outbreak in a Kangaroo care unit (where mothers nurse their premature babies) in Cape Town, South Africa confirmed their vulnerability [10]. Four out of six newborns developed pulmonary TB within 6 months after spending multiple days in the same room as a mother with undiagnosed pulmonary TB. Three of the four children had extensive disease at the time of diagnosis. In the absence of preventive measures ~50% of infants, even those delivered at term, developed active TB after infection [11].

In addition to their increased rate of progression to TB, infants are more likely to develop severe disseminated forms of TB associated with high morbidity and mortality. Before the availability of TB treatment, mortality varied from 55% in infants less than 6 months of age to 30% in those 1 to 2 years of age [12], and the death rate remains higher in infants than in other age groups even in the era of TB chemotherapy. Most lethal is congenital infection, which can occur either in utero or during birth; about one third of congenitally-infected children do not survive [13]. Fortunately, congenital TB infection is relatively rare; Schaaf reported that congenital TB constituted 1% of the childhood TB caseload in Tygerberg Children's Hospital in Cape Town [13]. More commonly, infants are infected postnatally; 8% of the pediatric TB caseload in Schaaf's report was comprised of such cases. Infants whose mothers have active TB are particularly at risk. The outcome of isolated pulmonary TB in infants is usually good if treatment is started early [13, 14], but they have increased susceptibility to disseminated (miliary) TB. Furthermore, TB meningitis becomes increasingly problematic after the first months of life. It has been estimated that, without prophylaxis, 30–40% of infants develop pulmonary disease following exposure, and a further 10–20% develop TB meningitis or disseminated (miliary) disease [11], which has near universal mortality in this age group [15, 16]. Most children who survive TB meningitis suffer from long-term sequelae including mental, motor, vision, and hearing impairment [17–19].

This paper provides an overview of immune factors that are likely to underlie the TB vulnerability of infants. Data from human infant studies will be discussed when available, and data from animal models will be utilized to provide deeper mechanistic insights. As the human immune system exhibits profound heterogeneity, probably only a subset of genetically susceptible individuals develop active TB. However, because of the age-specific maturation of the immune response, discussed further in this review, a larger proportion of infants seem to be susceptible. Environmental factors such as BCG vaccination and exposure to environmental mycobacteria may shape the infant immune response to *M.tb*, but knowledge in this area is limited, and we will restrict our discussion to factors intrinsic to the infant immune response. General reviews of immunity against *M.tb* and the immune status of infants are available [20–22]; we focus specifically on the intersection of these areas.

## 2. Immune Vulnerability of Infants to TB

The infant's immune system is shaped by past and present challenges encountered during life in the uterus and in the brave new postnatal world. Within the womb, detrimental immune responses between the mother and fetus are prevented by an intrauterine environment that restricts the development of cells that promote proinflammatory responses [23–25]. Birth represents a great transition from the sterile environment of the womb to a world full of bacteria; colonization of the newborn starts at the time of delivery. Several recent reviews describe the synergy between humans and bacteria [26–30], and the growing consensus is that the microbiome represents an essential part of our immune defense. The magnitude of colonization is impressive; a human adult is colonized with one kilogram of bacteria and in order for this beneficial process to occur, the immune system has to be tuned to allow it. The downside of this choice of nature is an infant's immune system that is susceptible to infections, and TB is arguably foremost among these. In the following section, we will first summarize the role of each relevant molecule or cell type in the immune response to TB, and then discuss how alterations during infancy may shape this response (summarized in Table 1).

### 2.1. Macrophages

#### 2.1.1. Role in TB

*M.tb* is transmitted by airborne particles when a person with pulmonary TB coughs [31]. Infection probably occurs in the distal alveoli where the bacteria are first ingested by alveolar macrophages. Macrophages have been shown to play a central role in the phagocytosis, growth arrest, and intracellular killing of *M.tb*. Combination of receptors and corresponding ligands is utilized for phagocytic entry into macrophages, and it has been hypothesized that the fate of *M.tb* depends on the initial receptors involved in the process [32, 33]. Once inside the phagosome, delivery of bactericidal lysosomal contents to the compartment will lead to the demise of the bacterium within. To avoid this fate, *M.tb* has the capacity to sustain the phagosome in its early, immature state [34, 35]. In addition to modulating the phagosome, recent studies suggest that *M.tb* can evade it all together by escaping into the cytoplasm [36, 37]. Ultimately, to kill or at least curb *M.tb* replication, macrophages must be activated by TNF and IFN-*γ* derived from antigen-specific T cells and perhaps innate cells. As the first responder to *M.tb* infection, the response of the alveolar macrophage is critical, not only for directly controlling TB, but also in setting the stage for the subsequent innate and adaptive immune responses [38]. Although T cell-mediated immunity is essential for immune control during later stages of disease, *M.tb*-specific T cells operate in large part by activating and equipping macrophages to control intracellular bacteria. Mouse studies have shown that during later stages of disease, monocyte-derived lung macrophage populations harbor a large percentage of *M.tb* ([39] and K. Urdahl, unpublished data).

#### 2.1.2. Status in Infants

Although the concentration of blood monocytes (precursors to tissue macrophages) in the fetus and neonate is on par with that in adults [40, 41], autopsy studies have revealed that full-term infants have very few detectable alveolar macrophages at birth [42]. In monkeys, these numbers increase to adult levels rapidly, within 1-2 days after birth [43], and cell recoveries from neonatal bronchoalveolar lavages suggest that a similar rapid increase takes place in human newborns [44]. Furthermore, chemotaxis of neonatal blood monocytes is slower than that of adult monocytes, which may further exacerbate the response to infection when lung resident macrophage numbers are low [45]. In addition to absolute numbers, the function of alveolar macrophages seems to be diminished in newborns. While neonatal blood monocytes demonstrate good phagocytic and microbicidal activity to a range of pathogens, neonatal alveolar macrophages perform these functions poorly [46–55]. For intracellular killing of *M.tb*, the ability to produce superoxide anion in response to IFN-*γ* is particularly important. Neonatal macrophages have a reduced capacity to perform this function, apparently due to decreased IFN-*γ* receptor signaling and diminished STAT-1 phosphorylation, despite comparable IFN-*γ* receptor expression [56]. The extent to which the paucity of alveolar macrophages and their limited functional capacity compromises immunity against TB during early infancy is unknown and deserves further study.

### 2.2. Neutrophils

#### 2.2.1. Role in TB

In the mouse model, neutrophils represent a transiently dominant population of infected lung cells in the second week of pulmonary TB infection [57], suggesting that neutrophils are the major population of lung phagocytes to acquire *M.tb* immediately after bacilli escape from alveolar macrophages. Whether or not neutrophils contribute to immunity by directly killing *M.tb* has not been well established. However, patients with chronic granulomatous disease that have impaired neutrophilic intracellular killing are susceptible to mycobacterial infections [58–61]. This correlation suggests that neutrophils may have a direct role in lysing *M.tb*, and a recent report in the zebrafish model of TB supports this idea [62]. Interestingly, in the mouse model, apoptotic neutrophils were recently shown to facilitate the uptake of *M.tb* by dendritic cells (DCs)—the third major phagocytic population to acquire *M.tb* in the lung [63]. Thus, neutrophils appear to be an important bridge between innate and adaptive immunity, as the acquisition of *M.tb* by DCs is essential for the initiation of the T cell response. Elimination of neutrophils slowed both DC acquisition of bacteria and *M.tb*-specific T cell priming [63].

#### 2.2.2. Status in Infants

Neonates, and in particular preterm neonates, have a decreased capacity to mobilize neutrophils in response to infection. This deficiency is in large part due to a limited neutrophil storage pool [64]. Further, neonatal neutrophils express low levels of integrins and selectins and perform poorly in functional assays for chemotaxis, rolling adhesion, transmigration, and lamellipodia formation, all of which are crucial for timely neutrophil recruitment to the site of infection [65–67]. The continuous production by neonatal monocytes of IL-6 (an effective neutrophil migration inhibitor) further depresses neutrophil function [68, 69]. Even the weaponry neutrophils have against microbial invaders is restricted, illustrated by reduced amounts of lactoferrin and diminished oxidase activity [64]. Although more investigation into the role of neutrophils in TB is needed, the profound deficiencies in neutrophil number and function during infancy have the potential to impair both innate and adaptive immunity to TB.

### 2.3. Dendritic Cells

#### 2.3.1. Role in TB

DCs comprise the third major phagocytic population to acquire *M.tb* in the lung. However, in contrast to neutrophils, the predominance of *M.tb*-infected DCs is long lived. In mice, *M.tb*-infected DCs first appear during the second week of infection and are abundant throughout the course of disease [39, 70]. In humans, DCs are also abundant in the tuberculous granuloma [71, 72]. Although the extent to which they kill or restrict intracellular growth of *M.tb* is unknown, mouse studies have shown that DCs play a critical role in the innate response to *M.tb* by producing cytokines essential for host defense. These cytokines include TNF, IL-1, and IL-12, and DCs are primary producers of the latter two [73, 74]. DCs are the consummate professional antigen-presenting cells and are essential for initiating and regulating the T cell response. To trigger *M.tb*-specific T cell responses, migratory DCs must first acquire *M.tb* in the lung, traffic to the lung draining lymph node, and present *M.tb* antigens to naïve antigen-specific T cells in the lymph node [39, 75–77]. DC populations that reside in lung granulomas are likely to be important for maintaining and regulating *M.tb*-specific T cells in the lung although this is less well studied than their role in the lymph node.

#### 2.3.2. Status in Infants

Although the number of resident DCs in the infant lung is unknown, neonates appear to have fewer circulating DCs than adults [78]. Furthermore, since most DCs in the lung that respond to *M.tb* infection are derived from blood monocytes, which have a diminished capacity for chemotaxis in neonates [45], it seems likely that the number of DCs responding to TB in the lung is lower in infants than in adults. As will be discussed further in a forthcoming section on pattern-recognition receptors in TB, infant DCs (as well as macrophages) also produce substantially lower levels of proinflammatory cytokines, including TNF, IL-1, and IL-12. The critical role of each of these cytokines in immunity against TB will be discussed in greater detail below. Studies examining the potential of neonatal DCs to process and present antigen to T cells provide conflicting results [22]. Although adult and neonatal blood DCs express similar cell surface levels of the MHC class II molecule HLA-DR as well as the costimulatory molecules CD40 and CD80, expression by neonatal DCs increases less in response to stimulation via toll-like receptors (TLRs) [79]. The diminished capacity of neonatal DCs to produce IL-12 may restrict their ability to prime Th1 cells, at least in some settings [22, 80]. As will be further discussed below, priming a rapid and robust Th1 response in the lung appears to be essential for immune control.

### 2.4. Cell Death Pathways

#### 2.4.1. Role in TB

Another way that *M.tb* shapes innate immunity is by manipulating macrophage death pathways. Necrosis and apoptosis can be observed simultaneously in tuberculous granulomas. Furthermore, both can be promoted by host and mycobacterial factors, and both can either promote or restrict bacterial replication, depending on the circumstances [20, 21]. During the early innate immune response, direct visualization of infected macrophages in the zebrafish embryo has shown that *M.tb* may orchestrate its own dissemination by inducing apoptosis through expression of virulence genes encoded by the RD1 region [81, 82]. The inflammatory response induced by *M.tb* attracts more macrophages that engulf apoptotic debris containing bacilli, and further intracellular bacterial replication ensues. Furthermore, some of the infected macrophages migrate to new sites where they attract additional uninfected macrophages and serve as niduses for new granuloma formation [81]. *M.tb* can also induce macrophage death by necrosis in some settings, for example through the induction of lipoxins and anti-inflammatory eicosanoids that suppress TNF production [21, 83]. One consequence of necrotic cell death is the release of extracellular mycobacteria, which are capable of even more exuberant growth in the extracellular milieu than they are within macrophages [20, 21]. Therefore, while apoptosis facilitates bacterial dissemination and necrosis promotes unchecked extracellular mycobacterial growth, optimal control of *M.tb* probably involves curbing mycobacterial replication within macrophages in a manner that does not induce cell death.

#### 2.4.2. Status in Infants

Infants do not usually form lung cavities with an abundance of extracellular bacteria as observed in adolescent and adult patients [11, 13]. Interestingly, lung cavities are occasionally seen in the absence of effective immune containment, in contrast to adolescents and adults where destructive immune responses contribute to cavity formation [84]. Future studies are needed to elucidate the mechanistic underpinnings of the scarcity of cavities in young children, and the possibility that macrophage cell death pathways are altered during infancy seems ripe for exploration. For example, diminished TNF-driven necrosis as a result of low TNF production by both innate and adaptive immune cells may provide a partial explanation. The fact that HIV-infected adults exhibit a similar phenotype [85–87] suggests that diminished T cell function (as discussed below) could be a contributing factor. Another intriguing hypothesis is that infant macrophages have a higher intrinsic predilection for apoptosis, a possibility that requires further investigation.

### 2.5. Pattern-Recognition Receptor Signaling

#### 2.5.1. Role in TB

*M.tb* components are recognized through multiple pattern-recognition receptors that trigger an inflammatory response. Important receptors include TLRs, cytosolic NOD like receptors (NLRs), C-type lectin receptors, and DC-SIGN [88–90]. Among the TLR family, TLR2, TLR4, and TLR9 play the most prominent roles in innate immunity to TB [90, 91]. TLR2 forms heterodimers with TLR1 or TLR6 and has been implicated in recognition of multiple mycobacterial cell wall glycolipids. TLR4 is activated by mycobacterial heat shock protein 60/65, whereas TLR9 recognizes unmethylated CpG motifs in bacterial DNA. Critical downstream cytokines induced by TLR- and NLR-mediated signals include the cytokines TNF, IL-1, and IL-12. Some pattern-recognition receptors also elicit an anti-inflammatory response in response to *M.tb*. The mannose receptor is a C-type lectin receptor expressed at high levels on alveolar macrophages. Man-LAM and other major components of the mycobacterial cell wall are natural ligands for the mannose receptor and their recognition suppresses IL-12 production [92–94]. DC-SIGN is expressed primarily on DCs. Engagement of DC-SIGN by mycobacterial components (including Man-LAM) in the presence of simultaneous TLR stimulation promotes an anti-inflammatory response, including IL-10 production [95].

#### 2.5.2. Status in Infants

Compared to adult myeloid cells, neonatal macrophages and DCs exhibit an altered pattern of TLR-mediated cytokine production, with decreased amounts of proinflammatory cytokines including TNF, IL-1, IL-6, and IL-12 and increased amounts of the anti-inflammatory cytokine IL-10 [96, 97]. Rather than simply reflecting an immature immune system, this probably represents a coping strategy to deal with the massive bacterial colonization that takes place during infancy, offering a means to avert a cytokine storm that might otherwise pose a serious inflammatory threat. The molecular mechanisms underlying this altered signaling are poorly understood, and in most cases, it is not clear if the altered responses are intrinsic to the signaling pathways or a result of suppression by extrinsic factors. Immune cells in human cord blood seem to express the same quantity of TLRs and downstream signaling molecules as those found in the peripheral blood of adults [98–100]. Reduced production of IL-12 in neonates has been correlated with the instability of a transcriptional complex that restricts induction of the IL-12p35 subunit [101]. On the other hand, extrinsic suppressive factors may play a role; for example, newborn plasma contains high concentrations of adenosine, which in turn causes high intracellular cAMP levels, that may enforce a bias against proinflammatory cytokine responses [96]. The distinct roles in the immune response to TB played by TNF, IL-1, IL-12, and IL-10, each differentially regulated in infants, will be discussed below. It is important to note that IL-6 also contributes to TB protection and exhibits altered expression levels during infancy [96, 97, 102]. Thus, although IL-6 may also play a role in the heightened vulnerability of infants to TB, it will not be discussed further in this review because little is currently known about its mechanistic mode of action during TB.

### 2.6. Tumor Necrosis Factor

#### 2.6.1. Role in TB

Central in the innate immune response by macrophages and DCs is their production of the inflammatory cytokine TNF, which plays multiple roles in immunity against TB [20–22]. Although the critical role of TNF was discovered in the mouse model of TB [103], its importance in human TB was subsequently verified by the finding that TNF blockade causes TB reactivation in latently infected individuals [104]. In addition to its role in activating macrophages to control intracellular *M.tb*, TNF is also important for chemokine production and recruitment of immune cells to the granuloma [105, 106]. Interestingly, TB disease in adolescents and adults is promoted in individuals genetically predisposed to produce either low or high amounts of TNF, whereas immune control is promoted by the production of intermediate TNF levels [107]. This confounding observation has been at least partially explained by the finding that both low and high TNF states lead to necrotic cell death of infected macrophages and enhanced extracellular bacillary growth, whereas intermediate TNF levels can curb intracellular bacterial growth without triggering macrophage necrosis [108, 109]. Importantly, corticosteroids that are routinely given in conjunction with antibiotics to all patients with TB meningitis, in an attempt to reduce inflammation-related sequelae, have recently been shown to benefit only those TB patients predisposed to overproduce TNF. In TB meningitis patients who produce low amounts of TNF, corticosteroids are not helpful and may even be detrimental [108].

#### 2.6.2. Status in Infants

TNF production by human neonatal macrophages and DCs is greatly diminished. Although the capacity to produce TNF gradually improves with age, adult levels of TNF production are not achieved until after one year of age [68, 96, 110]. While adolescents and adults may develop TB disease associated with exacerbated TNF production [107], it seems likely that most TB disease in infants is associated with a failure to produce enough TNF. This raises the question as to whether adjunctive steroid therapy should be given to infants with TB meningitis, because steroid therapy in adolescents and adults only benefits those with a propensity to produce high amounts of TNF [108]. Although several studies have demonstrated a benefit for steroids in TB meningitis in older children, adolescents, and adults [111], its utility in infants with TB meningitis has not been specifically addressed in a prospective trial. There is an urgent need to perform such a study because steroids are now routinely administered for all cases of TB meningitis, regardless of age. Given the inherent deficiency in TNF production in infants, it is plausible that infants might receive no benefit from this therapy, and may even be harmed.

### 2.7. Role of Other Cytokines Altered during Infancy

#### 2.7.1. IL-1

The finding that mice lacking MyD88 (the signaling adaptor molecule utilized by most membrane-bound TLR) are extremely susceptible to TB was originally interpreted to reflect the critical role of TLR for innate recognition of *M.tb* [112–114]. Mice lacking individual or combinations of specific TLR, however, exhibited much more subtle susceptibility phenotypes [115–117], probably reflecting the redundancy of pattern-recognition receptors that operate in TB. A role for IL-1 was then considered, because MyD88 also serves as the adapter protein for IL-1 receptor signaling, and TB susceptibility of mice lacking the IL-1 receptor is essentially identical to that of mice lacking MyD88 [118, 119]. Thus, IL-1 receptor signaling is sufficient to explain the requirement of MyD88 for TB resistance. Subsequent studies have implicated both IL-1 receptor-binding members of the IL-1 family (IL-1*α* and IL-1*β*) in contributing to immune resistance to TB [119, 120]. In mice, IL-1*α* and IL-1*β* play a dramatic role in immune protection, at least as great as any of the factors that are better appreciated to mediate TB resistance, including TNF, IL-12, and IFN-*γ*. A role for IL-1 in human immunity against TB is supported by several studies showing an association between polymorphisms in the IL-1 or IL-1 receptor genes and host resistance [121–124]. The mechanisms by which IL-1 mediates protection against TB are largely unknown. Although IL-1 can serve as a costimulatory molecule for T cells, particularly for the production of IL-17 [125], mice lacking IL-1 receptor signaling have profound susceptibility even before T cell responses are initiated [73, 74]. Thus, IL-1 plays an important innate role in TB, and the appearance of severe necrotic lung lesions in mice lacking IL-1-transmitted signals suggests that IL-1 may regulate cell death pathways crucial to TB pathogenesis [118, 119]. Recently, IL-1*β* production during later stages of mouse infection has been shown to be tightly regulated by IFN-*γ*-induced nitric oxide [126].

#### 2.7.2. IL-12

IL-12 is a heterodimeric molecule (IL-12p70) composed of the IL-12p40 and IL-12p35 subunits. It is produced by macrophages, and even more so by DCs, and its primary role in TB is to promote the expansion and maintenance of IFN-*γ*-producing T cells, which in turn activate macrophages to kill *M.tb*, or at least to curb its replication [127]. Mice or humans deficient in either IL-12 or the receptor through which it signals are extremely susceptible to TB disease [127–130]. IL-12 also has the potential to promote IFN-*γ* by innate cells, including *γδ* T cells, Natural killer (NK) cells, Natural killer T cells, CD1 group 1-restricted T cells-and the recently characterized mucosal-associated invariant T cells [131, 132], but further studies are needed to define the roles of these innate cell types in TB immunity.

#### 2.7.3. IL-10

IL-10 is an anti-inflammatory cytokine with pleiotropic immunoregulatory effects. Among these effects is its ability to restrict the differentiation of IFN-*γ*-producing T cells and to modulate IFN-*γ*-mediated signal transduction [133–135]. In mice, strong induction of IL-10 restricts protective immunity to TB, and in humans, a genetic polymorphism that results in enhanced innate production of IL-10 increases TB susceptibility [136, 137]. Despite its potential to negatively impact TB immunity, IL-10 probably also serves a host-protective role by limiting deleterious inflammatory damage to host tissues [138]. Thus, an optimal immune response to *M.tb* probably involves tightly regulated production of IL-10.

#### 2.7.4. Status of IL-1, IL-12, and IL-10 in Infants

IL-1 and IL-12 production is diminished in human neonates, whereas IL-10 production is greatly increased. Although production of proinflammatory cytokines gradually increases, adult capacity is not achieved until after one year of age for IL-1, and after two years of age for IL-12 [22, 80, 139–141]. Conversely, the production of IL-10 gradually decreases, but amounts comparable to those produced in adults are not seen until after one year of age [96, 110, 140].

### 2.8. Antimicrobial Peptides

#### 2.8.1. Role in TB

Antimicrobial peptides are produced and utilized by phagocytes and lung epithelial cells. Production of LL-37, one of the few bactericidal peptides that effectively kill *M.tb*,is upregulated in response to vitamin D and microbial interaction [142–144]. During *M.tb* infection, human beta defensin-2 (HBD2) is produced in the human lung by epithelial cells in response to IL-1 and TNF stimulation [145]. In addition to endogenous antimicrobials, human alveolar macrophages can utilize antimicrobial components obtained from cytotoxic T cells [146, 147] and apoptotic neutrophils alike [148]. Neutrophils alone carry a vast arsenal of antimicrobial peptides such as *α*-defensins, lactoferrin, cathelicidin, and lysozyme prepacked in granules [149]. These potent granular contents are utilized upon granule fusion with phagosomes [150] but may also be deployed directly to the outside of the phagocyte to fight extracellular pathogen or be delivered to macrophages. In addition to the direct mycobactericidal effects of antimicrobial peptides, some can also influence the chemotaxis of immature DCs and memory lymphocytes [151] and serve as a link between innate and acquired immune responses. Overall, these innate immune pathways restrict the unchecked replication of *M.tb* during the first few weeks of infection and also set the stage for an appropriate adaptive response, which is ultimately required for successful immune control of *M.tb*.

#### 2.8.2. Status in Infants

HBD2 and LL-37 are detectable in lung aspirates from healthy neonates and in increased amounts during pulmonary or systemic infections [152, 153]. However, the level of expression is low and *in vitro* stimulation of human fetal lung tissue with IL-1*β* and IFN-*γ* only induces a 2-fold increase in expression, in contrast to a 10-fold induction in adult tissue [152]. It is likely that the overall lack of IL-1*β*, IFN-*γ*, and TNF and general phagocyte inactivity combine to minimize the production of antibactericidal peptides, providing the newborn with reduced protection against pulmonary TB.

## 3. Adaptive Immunity

### 3.1. CD4 T Cells

#### 3.1.1. Role in TB

CD4 T cells are key antimycobacterial components of the adaptive immune response [20, 154–157], and TB is a leading cause of death in CD4 T cell lymphopenic HIV patient [7]. Key cytokines elaborated by protective Th1 effector cells include IFN-*γ* and TNF [20, 154–156] although CD4 T cells can also restrict *M.tb* replication by an IFN*γ*- and TNF-independent mechanism in mice [158]. Cytokines generated by Th2 cells, including IL-4 and IL-13, are detrimental to the protective response [159], partly by inhibiting autophagy-dependent killing of intracellular *M.tb* [160].

 Interestingly, the contribution of CD4 T cells in controlling TB may impact CD8 T cell function. Previous work has shown that CD4 T cell help is necessary for the induction of optimal CD8 T cell responses, particularly in the face of chronic infection, and CD8 T cells without CD4 T cell help lose effector function over time [161, 162]. This important role for CD4 T cells was originally discovered in mice [132, 161–164], but subsequently verified in nonhuman primates [165], and probably explains why HIV-infected humans with reduced numbers of CD4 T cells eventually lose CD8 T cell function [161, 162]. Such CD8 T cell exhaustion occurs gradually, with IL-2 production, cytolytic function, proliferative capacity lost at early stages, TNF production lost somewhat later, and IFN-*γ* production lost only very late [161–164]. The precise nature of CD4 T cell help for CD8 T cell responses is unclear, but possibilities include production of cytokines, including IL-2, licensing of antigen presenting cells, delivering survival signals, and controlling lymph node cellular input [166–168]. The relevance of these findings for TB has been demonstrated more recently. Maintenance of functional CD8 T cells in chronic mycobacterial infections in mice, including TB, requires help from CD4 T cells [169–171].

#### 3.1.2. Status in Infants

Altered infant CD4 T cell function has been widely described in both mice and humans. CD4 T cells from infants are characterized by poor proliferative capacity and diminished production of Th1 cytokines, including IFN-*γ* [172–175], as well as an increased propensity for Treg induction in the periphery [176, 177]. *In vitro* analyses of infant T cell responses have revealed clear differences in the intrinsic function of infant and adult T cells [178]. In fact, the Th2 bias of infant and neonatal CD4 T cells is reflected at the level of chromatin structure, as the Th2 cytokine loci in young mouse and human T cells is hypomethylated and poised for rapid transcription [179, 180]. An important factor explaining these intrinsic differences is that most T cells in infants are recent thymic emigrants (RTEs) [181, 182], those T cells that have recently completed thymic maturation and egress, whereas RTEs comprise only a small percentage of adult T cells [183]. CD4 RTEs are impaired in IL-2 and IFN-*γ* secretion and skewed to Th2 responses both *in vivo* and *in vitro* [181, 184–186]. Because of the important role of CD4 T cells in providing help to CD8 T cells, the diminished capacity of infant CD4 T cells likely restricts the function of CD8 T cells as well.

Despite these well-established biases of infant T cell responses, it is important to acknowledge that these tendencies are not absolute. Both BCG-immunized and *M.tb*-infected infants clearly mount a readily detectable population of IFN-*γ*-producing T cells [187, 188]. Future studies are needed to determine whether more subtle differences in their protective properties such as the kinetics of their expansion, tissue homing, longevity, or polyfunctionality may help to explain the immune vulnerability of infants to TB.

### 3.2. CD8 T Cells

#### 3.2.1. Role in TB

Appreciation of CD8 T cells as active components of the antimycobacterial adaptive immune response has come only recently [155]. CD8 T cells may be activated through cross presentation of mycobacterial antigens by DCs that have taken up apoptotic infected cells [189], but can also directly recognize *M.tb*-infected cells [190]. CD8 T cells help control *M.tb* replication by producing IFN-*γ*, as well as by perforin-mediated cytolysis of infected macrophages and direct killing of *M.tb* (reviewed in [155, 191]).

#### 3.2.2. Status in Infants

Mouse studies have shown that CD8 RTEs have a reduced capacity to mediate cytolysis and to produce IFN-*γ* [184, 192]. Consistent with these findings, CD8 T cells from human infants exhibit a reduced capacity to produce IFN-*γ* and granzyme B in the absence of exogenous IL-12 [193]. *In vivo* activated RTE-derived CD8 T cells are skewed to short-lived effector T cells and away from the memory precursor compartment [194]. Importantly, antigen-specific CD8 RTEs generate impaired memory T cells, demonstrating that if T cells first see antigen as RTEs, cell fate decisions are impacted long after [192].

### 3.3. Delayed Adaptive Immune Response

#### 3.3.1. Role in TB

Despite the essential role of IFN-*γ* producing T cells in immune protection against *M.tb*, the correlation between the magnitude of the IFN-*γ*-producing T cell response and the degree of protection is actually very poor. A large study of BCG-immunized infants recently revealed no correlation between protection and the frequency or cytokine expression profile of mycobacteria-specific T cells present in the blood [187]. Mouse studies have similarly shown no correlation between protection and IFN-*γ* producing T cell frequencies [195]; however, the rapidity by which *M.tb*-specific, IFN-*γ*-producing T cells reach the site of infection in the lung has been shown to be a critical predictor of protection [20, 196]. Unfortunately, the adaptive immune response to *M.tb* is significantly delayed compared to that observed in response to other pathogens [196]. Humans recently exposed to *M.tb* do not become tuberculin skin test positive until ~6 weeks after exposure [197, 198], whereas the T cell response to most pathogens peaks 1-2 weeks after exposure. Similarly, mice experimentally infected with *M.tb* via aerosol also exhibit a delayed T cell response that does not peak until several weeks after infection [20, 196], and the tractable nature of the mouse model has recently been utilized to dissect out many aspects of this delay. *M.tb*-specific T cell responses cannot be initiated until *M.tb* is ferried to the lung-draining lymph node by migratory DCs [39, 75]. This process does not occur until the second week of infection because *M.tb* replicates “under cover” in alveolar macrophages for a prolonged period [20, 196, 199]. *M.tb*-induced lipoxins contribute to this pathogen-induced loitering by suppressing apoptosis [200]. After their eventual release from alveolar macrophages, *M.tb* bacilli are phagocytized by and released from neutrophils before a significant number of bacteria are available for acquisition by DCs [201]. After *M.tb* is finally ferried to the lung draining lymph node by DCs, the effector T cell response is delayed even further by the expansion of pathogen-specific Treg cells at this site [202, 203]. *M.tb*-specific effector T cells do not reach the lung in sufficient numbers to begin to curb bacterial replication until three weeks after infection and take yet another week to peak in numbers [20, 196]. In the meantime, the delay enables *M.tb* to replicate exuberantly and establish a niche in the lung that facilitates high-burden chronic infection.

#### 3.3.2. Status in Infants

Whether adaptive immunity takes even longer to develop in infants than in older children and adults has not been formally studied. Clinical experience, however, suggests that this may be the case. Infants exposed to family members with active TB often take up to three months for detection of *M.tb*-specific, IFN-*γ*-producing T cells by a positive tuberculin skin test or IFN-*γ* release assay. The infant immune system has deficits in multiple factors that shape the rapidity of the adaptive immune response to TB. As previously discussed, infant macrophages, neutrophils, and DCs are present in low numbers and also have chemotactic deficits that impair their recruitment to sites of inflammation. These alterations have the potential to significantly delay the trafficking of *M.tb*-infected DCs to the lung-draining lymph node that is a prerequisite for initiating the *M.tb*-specific T cell response. Even after *M.tb* is delivered to the lymph node and T cell priming is initiated, effector T cell expansion may be slowed further by the increased propensity of infants to induce Treg responses [176, 177]. Taken together, it seems likely that infants have an even greater delay in the initiation of their adaptive immune response to TB than do older children and adults. Thus, infant T cells may not only be biased against making IFN-*γ*, but also against arriving in the lung in a timely manner.

Recent evidence shows that the T cell response of newborns immunized with BCG is quite delayed, peaking at 10 weeks after immunization [204]. This finding has important implications for the timing of an infant booster vaccine, which probably should not be administered until several weeks after this peak response [205]. Whether immunized adults would respond with different kinetics to BCG immunization is not known.

## 4. Practical Considerations

The vulnerability of infants to TB is profound and perhaps intractable. Nevertheless, there are several practical considerations. For perinatal TB, prevention is better than a cure (at least it is more easily achieved), and there are several opportunities to prevent neonatal *M.tb* infection and disease that are currently underutilized. Despite the fact that most neonatal TB is acquired from mothers with active disease, TB screening of women during the antenatal and postnatal period is often not adequately implemented in high-burden settings [206]. Such screening, however, could be readily integrated into primary antenatal care or (preventing mother-to-child transmission of HIV) PMTCT programs. Exposed newborns without signs of diseases should be offered TB prophylaxis. Even though TB diagnosis is difficult in infants, treatment should be started as early as possible to prevent complicated disease.

Infants in high TB endemic countries should be vaccinated with BCG, which is effective in preventing severe forms of disease. Given the enhanced susceptibility of neonates to mycobacterial infection, it seems a paradox that mycobacterium BCG is most effective when given to neonates. One possible explanation may be that BCG reaches a higher bacterial load for a prolonged period of time in infants, and this ultimately drives an enhanced antimycobacterial T cell memory response, despite the deficiencies in infant immunity outlined here. Another possibility is that infant effector T cells have an increased propensity to migrate to the uninfected lung [181, 184–186], which might play a dominant role in preventing progression to active TB. In any case, unraveling this conundrum will undoubtedly provide key insights into how long-lived immunity against TB can best be achieved and help inform the design of a desperately needed new and improved TB vaccine. Of interest, observational studies have suggested that BCG may also have a nonspecific beneficial effect on survival [207–209].

As highlighted in this review, TB is a fundamentally different disease in infants than in older children and adults. In older individuals, TB disease can be caused by an inappropriate and excessive inflammatory response to infection [107], whereas infant TB is a disease in which the inflammatory response is inadequate. Because the immune pathogenesis of infant TB is unique, TB immunotherapies that are beneficial for older individuals may be inappropriate for infants. As a possible case in point, adjunctive corticosteroid therapy for TB meningitis is currently routine for all ages, including infants. This practice stems from studies that have clearly shown modestly improved outcomes for TB meningitis in older children and adults receiving steroid adjunctive therapy [111]. The efficacy of steroids for infant TB meningitis, however, has not been specifically examined. Recently, the benefit of steroids for TB meningitis in adolescents and adults was found to be limited exclusively to a subset of individuals with a genetic propensity to produce high levels of TNF [108]. Steroids were not beneficial, and possibly detrimental, in individuals with lower capacities for TNF production. Because infants have an inherently restricted capacity to produce TNF, infants are unlikely to have TB meningitis that is caused by a hyperinflammatory state. Spurred by the growing problem of drug-resistant *M.tb*, potential immune-based TB therapies are gaining increased attention and research funding. Decisions to employ any TB immunotherapy in infants should not be made by extrapolating findings from studies of older individuals.

## 5. Summary

Inflammatory responses are dampened in infants, at least in part, to accommodate colonization with beneficial commensal bacteria. Unfortunately, key factors required for immune protection against TB are also down regulated. Macrophages, neutrophils, and DCs are present in low numbers and have impaired function. Their capacity to produce proinflammatory cytokines, including TNF, IL-1, and IL-12, is reduced, while their capacity to produce anti-inflammatory cytokines, including IL-10, is increased. Furthermore, infant T cells have an inherent bias against IFN-*γ* production, and favor production of cytokines such as IL-4 and IL-13 that are counterproductive during TB. Because of these immune alterations, infants are 5–10 times more likely to progress to active TB after infection and are also more likely to manifest severe, disseminated forms of disease. It is interesting to consider this immune dampening during infancy in light of the emerging idea that individuals susceptible to TB may represent both ends of the inflammatory spectrum; those that have a genetic predisposition to mount an inflammatory response to TB that is either too low or excessive may be susceptible, whereas those with a propensity for an intermediate response may be protected [108, 109]. If this principle proves to be generalizable for human TB, the reduced inflammatory response during infancy may shift some individuals that might otherwise be susceptible to hyperinflammatory TB into the protected zone, whereas many others that might otherwise be protected may become vulnerable due to their diminished capacity to mount an appropriate inflammatory response. The vulnerability of infants to TB and the unique nature of their immune response have practical implications for public health interventions, vaccine design, and immunotherapy.

## Figures and Tables

**Table 1:** Key elements of the immune response to TB with their activity in infants.

	Role in TB	Relative activity in infants
Macrophages	Intracellular **M.tb** killing and growth arrest [38, 210, 211]; alveolar macrophages initiate innate response [212]	Diminished chemotaxis [45] and intracellular killing [56]; reduced numbers of alveolar macrophages [40]

Neutrophils	Possible role in intracellular **M.tb** killing [62, 213–215]; promote T cell priming by facilitating **M.tb** uptake by DCs [63]	Diminished chemotaxis [65] and intracellular killing [64]; limited neutrophil storage pool [64]

Dendritic cells	Primary producers of IL-1 and IL-12 [73, 74]; initiate, regulate, and maintain T cell responses [39, 75–77, 216]	Low circulating number [78]; diminished capacity to produce TNF, IL-1, and IL-12 [80, 96, 217]; diminished capacity for priming Th1 cells

Cell death pathways	Regulate **M.tb** replication and dissemination [20, 21, 200, 218, 219]	Unknown, but necrotizing granulomas are unusual in infants

Pattern-recognition receptors	Phagocyte activation [88–90]; trigger cytokine production including TNF, IL-1, IL-12, and IL-10 [95]	Similar expression [98, 99], but altered signaling [100]; reduced triggering of proinflammatory and increased anti-inflammatory cytokines [97, 220]

TNF	Macrophage activation [20, 21, 103]; promotes immune cell recruitment [105, 106]; regulates cell death pathways [108, 109, 221]	Reduced levels and production capacity [68, 96, 110]

IL-1	Intracellular **M.tb** killing and/or growth arrest [118, 119]; T cell costimulation [125]	Reduced levels and production capacity [96, 222, 223]

IL-12	Induction and maintenance of IFN-*γ* producing T cells [20, 74, 154, 224, 225]	Reduced levels and production capacity [80, 139, 140]

IL-10	Restricts Th1 development and impairs IFN-*γ*-mediated signal transduction [133–135]	Increased levels and production capacity [96, 110, 140]

Antimicrobial peptides	Direct mycobactericidal activity [142, 143, 226]	Reduced levels and production capacity [152]

CD4^+^ T cells	Primary cellular source of IFN-*γ* and other factors that equip macrophages to restrict intracellular **M.tb** replication [20, 154, 227, 228]; provide help to maintain CD8^+^ effector T cells [169]	Bias against Th1 differentiation [172] and for Th2 and Treg induction [176]

CD8^+^ T cells	Cytolysis and production of IFN-*γ* [155, 190, 227]	Diminished IFN-*γ* production [193] and cytolytic function; bias towards short-lived effectors [192]

Delayed adaptive immune response	Facilitates prolonged mycobacterial replication and niche establishment in the lung [20, 196]	Infant immune status could even further delay the response

## References

[1] Dye C, Scheele S, Dolin P, Pathania V, Raviglione MC (1999). Global burden of tuberculosis: estimated incidence, prevalence, and mortality by country. *The Journal of the American Medical Association*.

[2] Nunn P, Williams B, Floyd K, Dye C, Elzinga G, Raviglione M (2005). Tuberculosis control in the era of HIV. *Nature Reviews Immunology*.

[3] Small PM, Fujiwara PI (2001). Management of tuberculosis in the United States. *The New England Journal of Medicine*.

[4] Lillebaek T, Dirksen A, Baess I, Strunge B, Thomsen VO, Andersen AB (2002). Molecular evidence of endogenous reactivation of *Mycobacterium tuberculosis* after 33 years of latent infection. *Journal of Infectious Diseases*.

[5] Van Rie A, Warren R, Richardson M (1999). Exogenous reinfection as a cause of recurrent tuberculosis after curative treatment. *The New England Journal of Medicine*.

[6] Charalambous S, Grant AD, Moloi V (2008). Contribution of reinfection to recurrent tuberculosis in South African gold miners. *International Journal of Tuberculosis and Lung Disease*.

[7] World Health Organization (2012). *Global Tuberculosis Report 2012*.

[8] Perez-Velez CM, Marais BJ (2012). Tuberculosis in children. *The New England Journal of Medicine*.

[9] UNICEF (2012). Committing to child survival: a promise renewed. *Progress Report*.

[10] Heyns L, Gie RP, Goussard P, Beyers N, Warren RM, Marais BJ (2006). Nosocomial transmission of *Mycobacterium tuberculosis* in kangaroo mother care units: a risk in tuberculosis-endemic areas. *Acta Paediatrica*.

[11] Marais BJ, Gie RP, Schaaf HS (2004). The natural history of childhood intra-thoracic tuberculosis: a critical review of literature from the pre-chemotherapy era. *International Journal of Tuberculosis and Lung Disease*.

[12] Lincoln EM (1950). Course and prognosis of tuberculosis in children. *The American Journal of Medicine*.

[13] Schaaf HS, Collins A, Bekker A, Davies PD (2010). Tuberculosis at extremes of age. *Respirology*.

[14] Goussard P, Gie RP, Kling S (2008). The outcome of infants younger than 6 months requiring ventilation for pneumonia caused by *Mycobacterium tuberculosis*. *Pediatric Pulmonology*.

[15] Seddon JA, Visser DH, Bartens M (2012). Impact of drug resistance on clinical outcome in children with tuberculous meningitis. *Pediatric Infectious Disease Journal*.

[16] Sharma SK, Mohan A, Sharma A (2012). Challenges in the diagnosis & treatment of miliary tuberculosis. *Indian Journal of Medical Research*.

[17] Donald PR, Schaaf HS, Schoeman JF (2005). Tuberculous meningitis and miliary tuberculosis: the rich focus revisited. *Journal of Infection*.

[18] van Well GTJ, Paes BF, Terwee CB (2009). Twenty years of pediatric tuberculous meningitis: a retrospective cohort study in the Western cape of South Africa. *Pediatrics*.

[19] Thwaites GE, Hien TT (2005). Tuberculous meningitis: many questions, too few answers. *The Lancet Neurology*.

[20] Cooper AM (2009). Cell-mediated immune responses in tuberculosis. *Annual Review of Immunology*.

[21] Ramakrishnan L (2012). Revisiting the role of the granuloma in tuberculosis. *Nature Reviews Immunology*.

[22] Remington JS, Klein JO, Wilson CB, Nizet V, Maldonado YA (2011). *Infectious Diseases of the Fetus and Newborn Infant*.

[23] Pasare C, Medzhitov R (2003). Toll pathway-dependent blockade of CD4^+^CD25^+^ T cell-mediated suppression by dendritic cells. *Science*.

[24] Firth MA, Shewen PE, Hodgins DC (2005). Passive and active components of neonatal innate immune defenses. *Animal Health Research Reviews*.

[25] Marodi L (2006). Innate cellular immune responses in newborns. *Clinical Immunology*.

[26] Tremaroli V, Backhed F (2012). Functional interactions between the gut microbiota and host metabolism. *Nature*.

[27] Costello EK, Stagaman K, Dethlefsen L, Bohannan BJ, Relman DA (2012). The application of ecological theory toward an understanding of the human microbiome. *Science*.

[28] Nicholson JK, Holmes E, Kinross J (2012). Host-gut microbiota metabolic interactions. *Science*.

[29] Johnson CL, Versalovic J (2012). The human microbiome and its potential importance to pediatrics. *Pediatrics*.

[30] Frei R, Lauener RP, Crameri R, O'Mahony L (2012). Microbiota and dietary interactions: an update to the hygiene hypothesis?. *Allergy*.

[31] Riley RL, Mills CC, O'Grady F, Sultan LU, Wittstadt F, Shivpuri DN (1962). Infectiousness of air from a tuberculosis ward. Ultraviolet irradiation of infected air: comparative infectiousness of different patients. *The American Review of Respiratory Disease*.

[32] Kang PB, Azad AK, Torrelles JB (2005). The human macrophage mannose receptor directs *Mycobacterium tuberculosis* lipoarabinomannan-mediated phagosome biogenesis. *Journal of Experimental Medicine*.

[33] Armstrong JA, D’Arcy Hart P (1975). Phagosome lysosome interactions in cultured macrophages infected with virulent tubercle bacilli. Reversal of the usual nonfusion pattern and observations on bacterial survival. *Journal of Experimental Medicine*.

[34] Hayakawa E, Tokumasu F, Nardone GA, Jin AJ, Hackley VA, Dvorak JA (2007). A *Mycobacterium tuberculosis*-derived lipid inhibits membrane fusion by modulating lipid membrane domains. *Biophysical Journal*.

[35] Perskvist N, Roberg K, Kulyté A, Stendahl O (2002). Rab5a GTPase regulates fusion between pathogen-containing phagosomes and cytoplasmic organelles in human neutrophils. *Journal of Cell Science*.

[36] van der Wel N, Hava D, Houben D (2007). *M. tuberculosis* and *M. leprae* translocate from the phagolysosome to the cytosol in myeloid cells. *Cell*.

[37] Simeone R, Bobard A, Lippmann J (2012). Phagosomal rupture by *Mycobacterium tuberculosis* results in toxicity and host cell death. *PLOS Pathogens*.

[38] Flynn JL, Chan J, Lin PL (2011). Macrophages and control of granulomatous inflammation in tuberculosis. *Mucosal Immunology*.

[39] Wolf AJ, Linas B, Trevejo-Nuñez GJ (2007). *Mycobacterium tuberculosis* infects dendritic cells with high frequency and impairs their function *in vivo*. *Journal of Immunology*.

[40] Weinberg AG, Rosenfeld CR, Manroe BL, Browne R (1985). Neonatal blood cell count in health and disease. II. Values for lymphocytes, monocytes, and eosinophils. *Journal of Pediatrics*.

[41] English BK, Hammond WP, Lewis DB, Brown CB, Wilson CB (1992). Decreased granulocyte-macrophage colony-stimulating factor production by human neonatal blood mononuclear cells and T cells. *Pediatric Research*.

[42] Alenghat E, Esterly JR (1984). Alveolar macrophages in perinatal infants. *Pediatrics*.

[43] Jacobs RF, Wilson CB, Smith AL, Haas JE (1983). Age-dependent effects of aminobutyryl muramyl dipeptide on alveolar macrophage function in infant and adult Macaca monkeys. *The American Review of Respiratory Disease*.

[44] Blahnik MJ, Ramanathan R, Riley CR, Minoo P (2001). Lipopolysaccharide-induced tumor necrosis factor-*α* and IL-10 production by lung macrophages from preterm and term neonates. *Pediatric Research*.

[45] Raghunathan R, Miller ME, Everett S, Leake RD (1982). Phagocyte chemotaxis in the perinatal period. *Journal of Clinical Immunology*.

[46] Becker ID, Robinson OM, Bazan TS (1981). Bactericidal capacity of newborn phagocytes against group B *β*-hemolytic streptococci. *Infection and Immunity*.

[47] Weston WL, Carson BS, Barkin RM, Slater GD, Dustin RD, Hecht SK (1977). Monocyte-macrophage function in the newborn. *The American Journal of Diseases of Children*.

[48] Kretschmer RR, Stewardson PB, Papierniak CK, Gotoff SP (1976). Chemotactic and bactericidal capacities of human newborn monocytes. *Journal of Immunology*.

[49] Orlowski JP, Sieger L, Anthony BF (1976). Bactericidal capacity of monocytes of newborn infants. *Journal of Pediatrics*.

[50] Hawes CS, Kemp AS, Jones WR (1980). *In vitro* parameters of cell-mediated immunity in the human neonate. *Clinical Immunology and Immunopathology*.

[51] Speer CP, Gahr M, Wieland M, Eber S (1988). Phagocytosis-associated functions in neonatal monocyte-derived macrophages. *Pediatric Research*.

[52] Conly ME, Speert DP (1991). Human neonatal monocyte-derived macrophages and neutrophils exhibit normal nonopsonic and opsonic receptor-mediated phagocytosis and superoxide anion production. *Biology of the Neonate*.

[53] D’Ambola JB, Sherman MP, Tashkin DP, Gong H (1988). Human and rabbit newborn lung macrophages have reduced anti-candida activity. *Pediatric Research*.

[54] Bellanti JA, Nerurkar LS, Zeligs BJ (1979). Host defenses in the fetus and neonate: studies of the alveolar macrophage during maturation. *Pediatrics*.

[55] Kurland G, Cheung ATW, Miller ME, Ayin SA, Cho MM, Ford EW (1988). The ontogeny of pulmonary defenses: alveolar macrophage function in neonatal and juvenile rhesus monkeys. *Pediatric Research*.

[56] Maródi L (2002). Deficient interferon-*γ* receptor-mediated signaling in neonatal macrophages. *Acta Paediatrica*.

[57] Blomgran R, Ernst JD (2011). Lung neutrophils facilitate activation of naive antigen-specific CD4^+^ T cells during *Mycobacterium tuberculosis* infection. *Journal of Immunology*.

[58] Doğru D, Kiper N, Özçelik U, Yalçin E, Tezcan I (2010). Tuberculosis in children with congenital immunodeficiency syndromes. *Tuberkuloz ve Toraks*.

[59] Lee PPW, Chan KW, Jiang L (2008). Susceptibility to mycobacterial infections in children with x-linked chronic granulomatous disease: a review of 17 patients living in a region endemic for tuberculosis. *Pediatric Infectious Disease Journal*.

[60] Movahedi M, Aghamohammadi A, Farhoudi A (2003). Respiratory manifestations of chronic granulomatous disease; a clinical survey of patients from Iranian primary immunodeficiency registry. *Iranian Journal of Allergy, Asthma and Immunology*.

[61] Reichenbach J, Rosenzweig S, Döffinger R, Dupuis S, Holland SM, Casanova JL (2001). Mycobacterial diseases in primary immunodeficiencies. *Current Opinion in Allergy and Clinical Immunology*.

[62] Yang CT, Cambier CJ, Davis JM, Hall CJ, Crosier PS, Ramakrishnan L (2012). Neutrophils exert protection in the early tuberculous granuloma by oxidative killing of mycobacteria phagocytosed from infected macrophages. *Cell Host and Microbe*.

[63] Blomgran R, Desvignes L, Briken V, Ernst JD (2012). *Mycobacterium tuberculosis* inhibits neutrophil apoptosis, leading to delayed activation of naive CD4 T cells. *Cell Host and Microbe*.

[64] Chirico G, Marconi M, de Amici M (1985). Deficiency of neutrophil bactericidal activity in term and preterm infants. A longitudinal study. *Biology of the Neonate*.

[65] Carr R (2000). Neutrophil production and function in newborn infants. *The British Journal of Haematology*.

[66] Urlichs F (2004). Neutrophil function in preterm and term infants. *NeoReviews*.

[67] Henneke P, Berner R (2006). Interaction of neonatal phagocytes with group B streptococcus: recognition and Response. *Infection and Immunity*.

[68] Angelone DF, Wessels MR, Coughlin M (2006). Innate immunity of the human newborn is polarized toward a high ratio of IL-6/TNF-*α* production *in vitro* and *in vivo*. *Pediatric Research*.

[69] Jones SA (2005). Directing transition from innate to acquired immunity: defining a role for IL-6. *Journal of Immunology*.

[70] Gonzalez-Juarrero M, Orme IM (2001). Characterization of murine lung dendritic cells infected with *Mycobacterium tuberculosis*. *Infection and Immunity*.

[71] Uehira K, Amakawa R, Ito T (2002). Dendritic cells are decreased in blood and accumulated in granuloma in tuberculosis. *Clinical Immunology*.

[72] Welsh KJ, Risin SA, Actor JK, Hunter RL (2011). Immunopathology of postprimary tuberculosis: increased T-regulatory cells and DEC-205-positive foamy macrophages in cavitary lesions. *Clinical and Developmental Immunology*.

[73] Mayer-Barber KD, Andrade BB, Barber DL (2011). Innate and adaptive interferons suppress IL-1*α* and IL-1*β* production by distinct pulmonary myeloid subsets during *Mycobacterium tuberculosis* infection. *Immunity*.

[74] Feng CG, Jankovic D, Kullberg M (2005). Maintenance of pulmonary Th1 effector function in chronic tuberculosis requires persistent IL-12 production. *Journal of Immunology*.

[75] Khader SA, Partida-Sanchez S, Bell G (2006). Interleukin 12p40 is required for dendritic cell migration and T cell priming after *Mycobacterium tuberculosis* infection. *Journal of Experimental Medicine*.

[76] Wolf AJ, Desvignes L, Linas B (2008). Initiation of the adaptive immune response to *Mycobacterium tuberculosis* depends on antigen production in the local lymph node, not the lungs. *Journal of Experimental Medicine*.

[77] Reiley WW, Calayag MD, Wittmer ST (2008). ESAT-6-specific CD4 T cell responses to aerosol *Mycobacterium tuberculosis* infection are initiated in the mediastinal lymph nodes. *Proceedings of the National Academy of Sciences of the United States of America*.

[78] Upham JW, Rate A, Rowe J, Kusel M, Sly PD, Holt PG (2006). Dendritic cell immaturity during infancy restricts the capacity to express vaccine-specific T-cell memory. *Infection and Immunity*.

[79] de Wit D, Tonon S, Olislagers V (2003). Impaired responses to toll-like receptor 4 and toll-like receptor 3 ligands in human cord blood. *Journal of Autoimmunity*.

[80] Kollmann TR, Crabtree J, Rein-Weston A (2009). Neonatal innate TLR-mediated responses are distinct from those of adults. *Journal of Immunology*.

[81] Davis JM, Ramakrishnan L (2009). The role of the granuloma in expansion and dissemination of early tuberculous infection. *Cell*.

[82] Ganguly N, Siddiqui I, Sharma P (2008). Role of *M. tuberculosis* RD-1 region encoded secretory proteins in protective response and virulence. *Tuberculosis*.

[83] Chen M, Divangahi M, Gan H (2008). Lipid mediators in innate immunity against tuberculosis: opposing roles of PGE2 and LXA4 in the induction of macrophage death. *Journal of Experimental Medicine*.

[84] Dempers J, Sens MA, Wadee SA, Kinney HC, Odendaal HJ, Wright CA (2011). Progressive primary pulmonary tuberculosis presenting as the sudden unexpected death in infancy: a case report. *Forensic Science International*.

[85] Barnes PF, Bloch AB, Davidson PT, Snider DE (1991). Current concepts: tuberculosis in patients with human immunodeficiency virus infection. *The New England Journal of Medicine*.

[86] Burman WJ, Jones BE (2003). Clinical and radiographic features of HIV-related tuberculosis. *Seminars in Respiratory Infections*.

[87] Sterling TR, Pham PA, Chaisson RE (2010). HIV infection-related tuberculosis: clinical manifestations and treatment. *Clinical Infectious Diseases*.

[88] Medzhitov R (2001). Toll-like receptors and innate immunity. *Nature Reviews Immunology*.

[89] Tallieux L, Schwartz O, Herrmann JL (2003). DC-SIGN is the major *Mycobacterium tuberculosis* receptor on human dendritic cells. *Journal of Experimental Medicine*.

[90] Kleinnijenhuis J, Oosting M, Joosten LAB, Netea MG, van Crevel R (2011). Innate immune recognition of *Mycobacterium tuberculosis*. *Clinical and Developmental Immunology*.

[91] Means TK, Wang S, Lien E, Yoshimura A, Golenbock DT, Fenton MJ (1999). Human toll-like receptors mediate cellular activation by *Mycobacterium tuberculosis*. *Journal of Immunology*.

[92] Kang BK, Schlesinger LS (1998). Characterization of mannose receptor-dependent phagocytosis mediated by *Mycobacterium tuberculosis* lipoarabinomannan. *Infection and Immunity*.

[93] Stein M, Keshav S, Harris N, Gordon S (1992). Interleukin 4 potently enhances murine macrophage mannose receptor activity: a marker of alternative immunologic macrophage activation. *Journal of Experimental Medicine*.

[94] Nigou J, Zelle-Rieser C, Gilleron M, Thurnher M, Puzo G (2001). Mannosylated lipoarabinomannans inhibit IL-12 production by human dendritic cells: evidence for a negative signal delivered through the mannose receptor. *Journal of Immunology*.

[95] Geijtenbeek TBH, van Vliet SJ, Koppel EA (2003). Mycobacteria target DC-SIGN to suppress dendritic cell function. *Journal of Experimental Medicine*.

[96] Kollmann TR, Levy O, Montgomery RR, Goriely S (2012). Innate immune function by toll-like receptors: distinct responses in newborns and the elderly. *Immunity*.

[97] Caron JE, La Pine TR, Augustine NH, Martins TB, Hill HR (2010). Multiplex analysis of toll-like receptor-stimulated neonatal cytokine response. *Neonatology*.

[98] Levy O, Zarember KA, Roy RM, Cywes C, Godowski PJ, Wessels MR (2004). Selective impairment of TLR-mediated innate immunity in human newborns: neonatal blood plasma reduces monocyte TNF-*α* induction by bacterial lipopeptides, lipopolysaccharide, and imiquimod, but preserves the response to R-848. *Journal of Immunology*.

[99] Strunk T, Currie A, Richmond P, Simmer K, Burgner D (2011). Innate immunity in human newborn infants: prematurity means more than immaturity. *Journal of Maternal-Fetal and Neonatal Medicine*.

[100] Yan SR, Qing G, Byers DM, Stadnyk AW, Al-Hertani W, Bortolussi R (2004). Role of MyD88 in diminished tumor necrosis factor *α* production by newborn mononuclear cells in response to lipopolysaccharide. *Infection and Immunity*.

[101] Aksoy E, Albarani V, Nguyen M (2007). Interferon regulatory factor 3-dependent responses to lipopolysaccharide are selectively blunted in cord blood cells. *Blood*.

[102] Ladel CH, Blum C, Dreher A, Reifenberg K, Kopf M, Kaufmann SHE (1997). Lethal tuberculosis in interleukin-6-deficient mutant mice. *Infection and Immunity*.

[103] Flynn JL, Goldstein MM, Chan J (1995). Tumor necrosis factor-*α* is required in the protective immune response against *Mycobacterium tuberculosis* in mice. *Immunity*.

[104] Keane J, Gershon S, Wise RP (2001). Tuberculosis associated with infliximab, a tumor necrosis factor *α*-neutralizing agent. *The New England Journal of Medicine*.

[105] Iliopoulos A, Psathakis K, Aslanidis S, Skagias L, Sfikakis PP (2006). Tuberculosis and granuloma formation in patients receiving anti-TNF therapy. *International Journal of Tuberculosis and Lung Disease*.

[106] Sedgwick JD, Riminton DS, Cyster JG, Körner H (2000). Tumor necrosis factor: a master-regulator of leukocyte movement. *Immunology Today*.

[107] Tobin DM, Vary JC, Ray JP (2010). The lta4h locus modulates susceptibility to mycobacterial infection in zebrafish and humans. *Cell*.

[108] Tobin DM, Roca FJ, Oh SF (2012). Host genotype-specific therapies can optimize the inflammatory response to mycobacterial infections. *Cell*.

[109] Roca FJ, Ramakrishnan L (2013). TNF dually mediates resistance and susceptibility to mycobacteria via mitochondrial reactive oxygen species. *Cell*.

[110] Burl S, Townend J, Njie-Jobe J (2011). Age-dependent maturation of toll-like receptor-mediated cytokine responses in gambian infants. *PLoS ONE*.

[111] Prasad K, Singh MB (2008). Corticosteroids for managing tuberculous meningitis. *Cochrane Database of Systematic Reviews*.

[112] Sugawara I, Yamada H, Mizuno S, Takeda K, Akira S (2003). Mycobacterial infection in MyD88-deficient mice. *Microbiology and Immunology*.

[113] Scanga CA, Bafica A, Feng CG, Cheever AW, Hieny S, Sher A (2004). MyD88-deficient mice display a profound loss in resistance to *Mycobacterium tuberculosis* associated with partially impaired Th1 cytokine and nitric oxide synthase 2 expression. *Infection and Immunity*.

[114] Fremond CM, Yeremeev V, Nicolle DM, Jacobs M, Quesniaux VF, Ryffel B (2004). Fatal *Mycobacterium tuberculosis* infection despite adaptive immune response in the absence of MyD88. *Journal of Clinical Investigation*.

[115] Feng CG, Scanga CA, Collazo-Custodio CM (2003). Mice lacking myeloid differentiation factor 88 display profound defects in host resistance and immune responses to *Mycobacterium avium* infection not exhibited by toll-like receptor 2 (TLR2)- and TLR4-deficient animals. *Journal of Immunology*.

[116] Bafica A, Scanga CA, Feng CG, Leifer C, Cheever A, Sher A (2005). TLR9 regulates Th1 responses and cooperates with TLR2 in mediating optimal resistance to *Mycobacterium tuberculosis*. *Journal of Experimental Medicine*.

[117] Hölscher C, Reiling N, Schaible UE (2008). Containment of aerogenic *Mycobacterium tuberculosis* infection in mice does not require MyD88 adaptor function for TLR2, -4 and -9. *European Journal of Immunology*.

[118] Fremond CM, Togbe D, Doz E (2007). IL-1 receptor-mediated signal is an essential component of MyD88-dependent innate response to *Mycobacterium tuberculosis* infection. *Journal of Immunology*.

[119] Mayer-Barber KD, Barber DL, Shenderov K (2010). Cutting edge: caspase-1 independent IL-1*β* production is critical for host resistance to *Mycobacterium tuberculosis* and does not require TLR signaling *in vivo*. *Journal of Immunology*.

[120] Cooper AM, Mayer-Barber KD, Sher A (2011). Role of innate cytokines in mycobacterial infection. *Mucosal Immunology*.

[121] Bellamy R, Ruwende C, Corrah T, McAdam KPWJ, Whittle HC, Hill AVS (1998). Assessment of the interleukin 1 gene cluster and other candidate gene polymorphisms in host susceptibility to tuberculosis. *Tubercle and Lung Disease*.

[122] Gomez LM, Camargo JF, Castiblanco J, Ruiz-Narváez EA, Cadena J, Anaya JM (2006). Analysis of IL1B, TAP1, TAP2 and IKBL polymorphisms on susceptibility to tuberculosis. *Tissue Antigens*.

[123] Awomoyi AA, Charurat M, Merchant A (2005). Polymorphism in IL1B: IL1B-511 association with tuberculosis and decreased lipopolysaccharide-induced IL-1*β* in IFN-*γ* primed ex-vivo whole blood assay. *Journal of Endotoxin Research*.

[124] Wilkinson RJ, Patel P, Llewelyn M (1999). Influence of polymorphism in the genes for the interleukin (IL)-1 receptor antagonist and IL-1*β* on tuberculosis. *Journal of Experimental Medicine*.

[125] Dungan LS, Mills KH (2011). Caspase-1-processed IL-1 family cytokines play a vital role in driving innate IL-17. *Cytokine*.

[126] Mishra BB, Rathinam VA, Martens GW (2013). Nitric oxide controls the immunopathology of tuberculosis by inhibiting NLRP3 inflammasome-dependent processing of IL-1*β*. *Nature Immunology*.

[127] Cooper AM, Solache A, Khader SA (2007). Interleukin-12 and tuberculosis: an old story revisited. *Current Opinion in Immunology*.

[128] Filipe-Santos O, Bustamante J, Chapgier A (2006). Inborn errors of IL-12/23- and IFN-*γ*-mediated immunity: molecular, cellular, and clinical features. *Seminars in Immunology*.

[129] Sahiratmadja E, Baak-Pablo R, de Visser AW (2007). Association of polymorphisms in IL-12/IFN-*γ* pathway genes with susceptibility to pulmonary tuberculosis in Indonesia. *Tuberculosis*.

[130] Altare F, Durandy A, Lammas D (1998). Impairment of mycobacterial immunity in human interleukin-12 receptor deficiency. *Science*.

[131] Philips JA, Ernst JD (2012). Tuberculosis pathogenesis and immunity. *Annual Review of Pathology*.

[132] Gold MC, Lewinsohn DM (2013). Co-dependents: MR1-restricted MAIT cells and their antimicrobial function. *Nature Reviews Microbiology*.

[133] Fiorentino DF, Zlotnik A, Vieira P (1991). IL-10 acts on the antigen-presenting cell to inhibit cytokine production by Th1 cells. *Journal of Immunology*.

[134] Beamer GL, Flaherty DK, Assogba BD (2008). Interleukin-10 promotes *Mycobacterium tuberculosis* disease progression in CBA/J mice. *Journal of Immunology*.

[135] Fiorentino DF, Zlotnik A, Mosmann TR, Howard M, O’Garra A (1991). IL-10 inhibits cytokine production by activated macrophages. *Journal of Immunology*.

[136] Awomoyi AA, Marchant A, Howson JMM, McAdam KPWJ, Blackwell JM, Newport MJ (2002). Interleukin-10, polymorphism in SLC11A1 (formerly NRAMP1), and susceptibility to tuberculosis. *Journal of Infectious Diseases*.

[137] Turner J, Gonzalez-Juarrero M, Ellis DL (2002). *In vivo* IL-10 production reactivates chronic pulmonary tuberculosis in C57BL/6 mice. *Journal of Immunology*.

[138] Higgins DM, Sanchez-Campillo J, Rosas-Taraco AG, Lee EJ, Orme IM, Gonzalez-Juarrero M (2009). Lack of IL-10 alters inflammatory and immune responses during pulmonary *Mycobacterium tuberculosis* infection. *Tuberculosis*.

[139] Belderbos ME, van Bleek GM, Levy O (2009). Skewed pattern of toll-like receptor 4-mediated cytokine production in human neonatal blood: low LPS-induced IL-12p70 and high IL-10 persist throughout the first month of life. *Clinical Immunology*.

[140] Corbett NP, Blimkie D, Ho KC (2010). Ontogeny of toll-like receptor mediated cytokine responses of human blood mononuclear cells. *PLoS ONE*.

[141] Upham JW, Lee PT, Holt BJ (2002). Development of interleukin-12-producing capacity throughout childhood. *Infection and Immunity*.

[142] Liu PT, Stenger S, Li H (2006). Toll-like receptor triggering of a vitamin D-mediated human antimicrobial response. *Science*.

[143] Martineau AR, Newton SM, Wilkinson KA (2007). Neutrophil-mediated innate immune resistance to mycobacteria. *Journal of Clinical Investigation*.

[144] Rivas-Santiago B, Sada E, Tsutsumi V, Aguilar-Léon D, Contreras JL, Hernández-Pando R (2006). *β*-defensin gene expression during the course of experimental tuberculosis infection. *Journal of Infectious Diseases*.

[145] Rivas-Santiago B, Schwander SK, Sarabia C (2005). Human *β*-defensin 2 is expressed and associated with *Mycobacterium tuberculosis* during infection of human alveolar epithelial cells. *Infection and Immunity*.

[146] Stenger S, Hanson DA, Teitelbaum R (1998). An antimicrobial activity of cytolytic T cells mediated by granulysin. *Science*.

[147] Ochoa MT, Stenger S, Sieling PA (2001). T-cell release of granulysin contributes to host defense in leprosy. *Nature Medicine*.

[148] Tan BH, Meinken C, Bastian M (2006). Macrophages acquire neutrophil granules for antimicrobial activity against intracellular pathogens. *Journal of Immunology*.

[149] Faurschou M, Borregaard N (2003). Neutrophil granules and secretory vesicles in inflammation. *Microbes and Infection*.

[150] Segal AW (2005). How neutrophils kill microbes. *Annual Review of Immunology*.

[151] Yang D, Biragyn A, Kwak LW, Oppenheim JJ (2002). Mammalian defensins in immunity: more than just microbicidal. *Trends in Immunology*.

[152] Starner TD, Agerberth B, Gudmundsson GH, McCray PB (2005). Expression and activity of *β*-defensins and LL-37 in the developing human lung. *Journal of Immunology*.

[153] Schaller-Bals S, Schulze A, Bals R (2002). Increased levels of antimicrobial peptides in tracheal aspirates of newborn infants during infection. *The American Journal of Respiratory and Critical Care Medicine*.

[154] Flynn JL, Chan J (2001). Immunology of tuberculosis. *Annual Review of Immunology*.

[155] Woodworth JSM, Behar SM (2006). *Mycobacterium tuberculosis*-specific CD8^+^ T cells and their role in immunity. *Critical Reviews in Immunology*.

[156] Kagina BMN, Abel B, Bowmaker M (2009). Delaying BCG vaccination from birth to 10 weeks of age may result in an enhanced memory CD4 T cell response. *Vaccine*.

[157] Elliott AM, Hodsdon WS, Kyosiimire J (2004). Cytokine responses and progression to active tuberculosis in HIV-1-infected Ugandans: a prospective study. *Transactions of the Royal Society of Tropical Medicine and Hygiene*.

[158] Gallegos AM, van Heijst JWJ, Samstein M, Su X, Pamer EG, Glickman MS (2011). A *γ* interferon independent mechanism of CD4 T cell mediated control of *M. tuberculosis* infection *in vivo*. *PLoS Pathogens*.

[159] Dorhoi A, Reece ST, Kaufmann SHE (2011). For better or for worse: the immune response against *Mycobacterium tuberculosis* balances pathology and protection. *Immunological Reviews*.

[160] Harris J, de Haro SA, Master SS (2007). T helper 2 cytokines inhibit autophagic control of intracellular *Mycobacterium tuberculosis*. *Immunity*.

[161] Kalams SA, Walker BD (1998). The critical need for CD4 help in maintaining effective cytotoxic T lymphocyte responses. *Journal of Experimental Medicine*.

[162] Shin H, Wherry EJ (2007). CD8 T cell dysfunction during chronic viral infection. *Current Opinion in Immunology*.

[163] Matloubian M, Concepcion RJ, Ahmed R (1994). CD4^+^ T cells are required to sustain CD8^+^ cytotoxic T-cell responses during chronic viral infection. *Journal of Virology*.

[164] Fuller MJ, Khanolkar A, Tebo AE, Zajac AJ (2004). Maintenance, loss, and resurgence of T cell responses during acute, protracted, and chronic viral infections. *Journal of Immunology*.

[165] Grakoui A, Shoukry NH, Woollard DJ (2003). HCV persistence and immune evasion in the absence of memory T cell help. *Science*.

[166] Bevan MJ (2004). Helping the CD8^+^ T-cell response. *Nature Reviews Immunology*.

[167] Zhang S, Zhang H, Zhao J (2009). The role of CD4 T cell help for CD8 CTL activation. *Biochemical and Biophysical Research Communications*.

[168] Kumamoto Y, Mattei LM, Sellers S, Payne GW, Iwasaki A (2011). CD4^+^ T cells support cytotoxic T lymphocyte priming by controlling lymph node input. *Proceedings of the National Academy of Sciences of the United States of America*.

[169] Bold TD, Ernst JD (2012). CD4^+^ T cell-dependent IFN-*γ* production by CD8^+^ effector T cells in *Mycobacterium tuberculosis* infection. *Journal of Immunology*.

[170] Bold TD, Ernst JD (2012). CD4^+^ T cell-dependent IFN*γ* production by CD8^+^ effector T cells in *Mycobacterium tuberculosis* infection. *Journal of Immunology*.

[171] Dudani R, Chapdelaine Y, van Faassen H (2002). Multiple mechanisms compensate to enhance tumor-protective CD8^+^ T cell response in the long-term despite poor CD8^+^ T cell priming initially: comparison between an acute versus a chronic intracellular bacterium expressing a model antigen. *Journal of Immunology*.

[172] Adkins B, Leclerc C, Marshall-Clarke S (2004). Neonatal adaptive immunity comes of age. *Nature Reviews Immunology*.

[173] Siegrist CA, Aspinall R (2009). B-cell responses to vaccination at the extremes of age. *Nature Reviews Immunology*.

[174] Zaghouani H, Hoeman CM, Adkins B (2009). Neonatal immunity: faulty T-helpers and the shortcomings of dendritic cells. *Trends in Immunology*.

[175] PrabhuDas M, Adkins B, Gans H (2011). Challenges in infant immunity: implications for responses to infection and vaccines. *Nature Immunology*.

[176] Wang G, Miyahara Y, Guo Z, Khattar M, Stepkowski SM, Chen W (2010). ‘Default’ generation of neonatal regulatory T cells. *Journal of Immunology*.

[177] Mold JE, Venkatasubrahmanyam S, Burt TD (2010). Fetal and adult hematopoietic stem cells give rise to distinct T cell lineages in humans. *Science*.

[178] Belderbos M, Levy O, Bont L (2009). Neonatal innate immunity in allergy development. *Current Opinion in Pediatrics*.

[179] Rose S, Lichtenheld M, Foote MR, Adkins B (2007). Murine neonatal CD4^+^ cells are poised for rapid Th2 effector-like function. *Journal of Immunology*.

[180] Webster RB, Rodriguez Y, Klimecki WT, Vercelli D (2007). The human IL-13 locus in neonatal CD4^+^ T cells is refractory to the acquisition of a repressive chromatin architecture. *The Journal of Biological Chemistry*.

[181] Haines CJ, Giffon TD, Lu LS (2009). Human CD4^+^ T cell recent thymic emigrants are identified by protein tyrosine kinase 7 and have reduced immune function. *Journal of Experimental Medicine*.

[182] Lewis DB, Haines C, Ross D (2011). Protein tyrosine kinase 7: a novel surface marker for human recent thymic emigrants with potential clinical utility. *Journal of Perinatology*.

[183] Olsen NJ, Kovacs WJ (2011). Evidence that androgens modulate human thymic T cell output. *Journal of Investigative Medicine*.

[184] Boursalian TE, Golob J, Soper DM, Cooper CJ, Fink PJ (2004). Continued maturation of thymic emigrants in the periphery. *Nature Immunology*.

[185] Opiela SJ, Koru-Sengul T, Adkins B (2009). Murine neonatal recent thymic emigrants are phenotypically and functionally distinct from adult recent thymic emigrants. *Blood*.

[186] Hendricks DW, Fink PJ (2011). Recent thymic emigrants are biased against the T-helper type 1 and toward the T-helper type 2 effector lineage. *Blood*.

[187] Kagina BMN, Abel B, Scriba TJ (2010). Specific T cell frequency and cytokine expression profile do not correlate with protection against tuberculosis after bacillus Calmette-Guérin vaccination of newborns. *The American Journal of Respiratory and Critical Care Medicine*.

[188] Lewinsohn DA, Zalwango S, Stein CM (2008). Whole blood interferon-*γ* responses to *Mycobacterium tuberculosis* antigens in young household contacts of persons with tuberculosis in Uganda. *PLoS ONE*.

[189] Schaible UE, Winau F, Sieling PA (2003). Apoptosis facilitates antigen presentation to T lymphocytes through MHC-I and CD1 in tuberculosis. *Nature Medicine*.

[190] Lewinsohn DM, Grotzke JE, Heinzel AS (2006). Secreted proteins from *Mycobacterium tuberculosis* gain access to the cytosolic MHC class-I antigen-processing pathway. *Journal of Immunology*.

[191] Kaufmann SHE (2010). Future vaccination strategies against tuberculosis: thinking outside the box. *Immunity*.

[192] Makaroff LE, Hendricks DW, Niec RE, Fink PJ (2009). Postthymic maturation influences the CD8 T cell response to antigen. *Proceedings of the National Academy of Sciences of the United States of America*.

[193] Mccarron MJ, Reen DJ (2010). Neonatal CD8^+^ T-cell differentiation is dependent on interleukin-12. *Human Immunology*.

[194] Joshi NS, Kaech SM (2008). Effector CD8 T cell development: a balancing act between memory cell potential and terminal differentiation. *Journal of Immunology*.

[195] Mittrücker HW, Steinhoff U, Köhler A (2007). Poor correlation between BCG vaccination-induced T cell responses and protection against tuberculosis. *Proceedings of the National Academy of Sciences of the United States of America*.

[196] Urdahl KB, Shafiani S, Ernst JD (2011). Initiation and regulation of T-cell responses in tuberculosis. *Mucosal Immunology*.

[197] Wallgren A (1948). The time-table of tuberculosis. *Tubercle*.

[198] Poulsen A (1950). Some clinical features of tuberculosis. 1. Incubation period. *Acta tuberculosea Scandinavica*.

[199] Behar SM, Divangahi M, Remold HG (2010). Evasion of innate immunity by *Mycobacterium tuberculosis*: is death an exit strategy?. *Nature Reviews Microbiology*.

[200] Divangahi M, Desjardins D, Nunes-Alves C, Remold HG, Behar SM (2010). Eicosanoid pathways regulate adaptive immunity to *Mycobacterium tuberculosis*. *Nature Immunology*.

[201] Ernst JD (2012). The immunological life cycle of tuberculosis. *Nature Reviews Immunology*.

[202] Shafiani S, Tucker-Heard G, Kariyone A, Takatsu K, Urdahl KB (2010). Pathogen-specific regulatory T cells delay the arrival of effector T cells in the lung during early tuberculosis. *Journal of Experimental Medicine*.

[203] Shafiani S, Dinh C, Ertelt JM Expansion and subsequent culling of pathogen-specific regulatory T cells during *Mycobacterium tuberculosis* infection.

[204] Soares AP, Chung CKK, Choice T (2013). Longitudinal changes in CD4^+^ T-cell memory responses induced by BCG vaccination of newborns. *Journal of Infectious Diseases*.

[205] Sallusto F, Lanzavecchia A, Araki K, Ahmed R (2010). From vaccines to memory and back. *Immunity*.

[206] Bekker A, Preez KD, Schaaf HS, Cotton MF, Hesseling AC (2012). High tuberculosis exposure among neonates in a high tuberculosis and human immunodeficiency virus burden setting. *International Journal of Tuberculosis and Lung Disease*.

[207] Roth A, Jensen H, Garly ML (2004). Low birth weight infants and Calmette-Guérin bacillus vaccination at birth: community study from Guinea-Bissau. *Pediatric Infectious Disease Journal*.

[208] Garly ML, Martins CL, Balé C (2003). BCG scar and positive tuberculin reaction associated with reduced child mortality in West Africa: a non-specific beneficial effect of BCG?. *Vaccine*.

[209] Aaby P, Roth A, Ravn H (2011). Randomized trial of BCG vaccination at birth to low-birth-weight children: beneficial nonspecific effects in the neonatal period?. *Journal of Infectious Diseases*.

[210] Schlesinger LS (1996). Entry of *Mycobacterium tuberculosis* into mononuclear phagocytes. *Current Topics in Microbiology and Immunology*.

[211] Pieters J (2008). *Mycobacterium tuberculosis* and the macrophage: maintaining a balance. *Cell Host and Microbe*.

[212] Martin CJ, Booty MG, Rosebrock TR (2012). Efferocytosis is an innate antibacterial mechanism. *Cell Host and Microbe*.

[213] Eruslanov EB, Lyadova IV, Kondratieva TK (2005). Neutrophil responses to *Mycobacterium tuberculosis* infection in genetically susceptible and resistant mice. *Infection and Immunity*.

[214] Eum SY, Kong JH, Hong MS (2010). Neutrophils are the predominant infected phagocytic cells in the airways of patients with active pulmonary TB. *Chest*.

[215] Lowe DM, Redford PS, Wilkinson RJ, 'Garra A O, Martineau AR (2012). Neutrophils in tuberculosis: friend or foe?. *Trends in Immunology*.

[216] Tian T, Woodworth J, Sköld M, Behar SM (2005). *In vivo* depletion of CD11c^+^ cells delays the CD4^+^ T cell response to *Mycobacterium tuberculosis* and exacerbates the outcome of infection. *Journal of Immunology*.

[217] de Wit D, Olislagers V, Goriely S (2004). Blood plasmacytoid dendritic cell responses to CpG oligodeoxynucleotides are impaired in human newborns. *Blood*.

[218] Behar SM, Martin CJ, Booty MG (2011). Apoptosis is an innate defense function of macrophages against *Mycobacterium tuberculosis*. *Mucosal Immunology*.

[219] Kelly DM, ten Bokum AMC, O’Leary SM, O’Sullivan MP, Keane J (2008). Bystander macrophage apoptosis after *Mycobacterium tuberculosis* H37Ra infection. *Infection and Immunity*.

[220] Lotz M, Gütle D, Walther S, Ménard S, Bogdan C, Hornef MW (2006). Postnatal acquisition of endotoxin tolerance in intestinal epithelial cells. *Journal of Experimental Medicine*.

[221] Miller EA, Ernst JD (2008). Illuminating the black box of TNF action in tuberculous granulomas. *Immunity*.

[222] Nguyen M, Leuridan E, Zhang T (2010). Acquisition of adult-like TLR4 and TLR9 responses during the first year of life. *PLoS ONE*.

[223] Lisciandro JG, Prescott SL, Nadal-Sims MG (2012). Ontogeny of toll-like and NOD-like receptor-mediated innate immune responses in Papua New Guinean infants. *PLoS ONE*.

[224] Alcaïs A, Fieschi C, Abel L, Casanova JL (2005). Tuberculosis in children and adults: two distinct genetic diseases. *Journal of Experimental Medicine*.

[225] Alcais A, Quintana-Murci L, Thaler DS, Schurr E, Abel L, Casanova JL (2010). Life-threatening infectious diseases of childhood: single-gene inborn errors of immunity?. *Annals of the New York Academy of Sciences*.

[226] Castañeda-Delgado J, Hernández-Pando R, Serrano CJ (2010). Kinetics and cellular sources of cathelicidin during the course of experimental latent tuberculous infection and progressive pulmonary tuberculosis. *Clinical and Experimental Immunology*.

[227] North RJ, Jung YJ (2004). Immunity to tuberculosis. *Annual Review of Immunology*.

[228] Gutierrez MG, Master SS, Singh SB, Taylor GA, Colombo MI, Deretic V (2004). Autophagy is a defense mechanism inhibiting BCG and *Mycobacterium tuberculosis* survival in infected macrophages. *Cell*.

